# Confidence in the efficacy and safety of dietary supplements among United States active duty army personnel

**DOI:** 10.1186/1472-6882-12-182

**Published:** 2012-10-10

**Authors:** Christina E Carvey, Emily K Farina, Harris R Lieberman

**Affiliations:** 1 Military Nutrition Division, U.S, Army Research Institute of Environmental Medicine, Kansas Street, Natick, MA, 01760, USA

**Keywords:** Consumer beliefs, Military, Government regulation, Dietary supplement health and education act (DSHEA)

## Abstract

**Background:**

United States Army Soldiers regularly use dietary supplements (DS) to promote general health, enhance muscle strength, and increase energy, but limited scientific evidence supports the use of many DS for these benefits. This study investigated factors associated with Soldiers’ confidence in the efficacy and safety of DS, and assessed Soldiers’ knowledge of federal DS regulatory requirements.

**Methods:**

Between 2006 and 2007, 990 Soldiers were surveyed at 11 Army bases world-wide to assess their confidence in the effectiveness and safety of DS, knowledge of federal DS regulations, demographic characteristics, lifestyle-behaviors and DS use.

**Results:**

A majority of Soldiers were at least somewhat confident that DS work as advertised (67%) and thought they are safe to consume (71%). Confidence in both attributes was higher among regular DS users than non-users. Among users, confidence in both attributes was positively associated with rank, self-rated diet quality and fitness level, education, and having never experienced an apparent DS-related adverse event. Fewer than half of Soldiers knew the government does not require manufacturers to demonstrate efficacy, and almost a third incorrectly believed there are effective pre-market federal safety requirements for DS.

**Conclusions:**

Despite limited scientific evidence supporting the purported benefits and safety of many popular DS, most Soldiers were confident that DS are effective and safe. The positive associations between confidence and DS use should be considered when developing DS-related interventions or policies. Additionally, education to clarify Soldiers’ misperceptions about federal DS safety and efficacy regulations is warranted.

## Background

Since the passage of the Dietary Supplements Health and Education Act (DSHEA) in 1994, U.S. sales of dietary supplements (DS) – defined by the legislation as products intended to supplement the diet, including vitamins, minerals, herbs and botanicals, amino acids, and substances such as enzymes, organ tissues, glandulars, and metabolites [1] – have risen dramatically from $8.8 billion [2] to an estimated $28.7 billion for 2010 [3]. There has also been a substantial increase in the proportion of adults, both civilian and military, who regularly use DS – current estimates suggest that 52% of U.S. adults and 53% of military personnel regularly use some form of DS [4,5].

Dietary supplements are commonly consumed by Americans to promote general health, improve energy or memory, and to treat or prevent medical conditions such as osteoporosis or arthritis [6]. However, for a majority of supplements, there is limited evidence to support such benefits. Consumers may also believe that DS are “natural” remedies, and are, therefore, safer to consume than traditional medical treatments, such as drugs [7]. However, U.S. federal regulations do not subject DS to the same stringent safety and efficacy regulations that the Food and Drug Administration (FDA) imposes on prescription and over-the-counter drugs [8].

Although manufacturers are legally responsible for ensuring the safety of DS and for ensuring any product claims are not false or misleading [1], they are not required to provide definitive pre-market substantiation of either safety or efficacy, or to have the product evaluated by an independent scientific regulatory entity. Rather, the onus for determining if or whether a DS is unsafe is on the FDA; for the agency to recall a supplement, it must obtain sufficient evidence that the specific supplement in question is unsafe and poses a “significant or unreasonable risk of illness or injury” [8,9]. Manufacturers must inform the FDA prior to introducing a new dietary ingredient to the market. However this notification is often not accompanied by a safety assessment of the product [10]. Statements and claims suggesting possible benefits of consuming a DS are also minimally regulated. Manufacturers may make “structure-function claims” on packaging, provided claims do not reference a specific disease or condition, and provided their claims are qualified with the disclaimer, “This statement has not been evaluated by the Food and Drug Administration. This product is not intended to diagnose, treat, cure, or prevent any disease”.

Industry data indicate that consumer confidence in the safety, quality and effectiveness of DS has increased over the past decade. In 2001, 74% of American adults surveyed indicated they were somewhat or very confident in the safety, quality and effectiveness of dietary supplements. By 2010, that number had increased to 82% [11,12]. This high level of confidence may be due to consumers’ misconceptions about the extent of pre-market review and regulatory oversight that a DS must undergo. Many Americans are unaware or misinformed about the FDA’s role in regulating DS [7,9,13,14], and may assume that DS are subject to the same efficacy and safety testing as OTC drugs [8]. Such beliefs may foster a false sense of security in the efficacy and safety of supplements. In fact, making individuals explicitly aware that the FDA had not approved a particular DS made them more skeptical of the product’s safety, although it did not affect participants’ ratings of product efficacy [15]. This may suggest consumers are willing to rely on their own experiences to form opinions regarding DS efficacy, but are less likely to rely on their own experiences to form opinions regarding DS safety.

While there is an increasing body of literature examining characteristics of supplement users, relatively little is known about the factors that influence consumer confidence in DS. However, regular users of DS are more inclined to believe supplements are effective and safe compared to non-users [2,9,16,17]. The purpose of this study was to assess beliefs about DS efficacy and safety among U.S. Army Active Duty personnel, a population known to have a high frequency of DS use [4], and to investigate whether certain demographic and lifestyle factors of DS users are associated with higher confidence in either attribute. We hypothesized that confidence in DS efficacy and safety would be associated with age, education, and self-reported fitness level because similar factors were associated with DS use among military personnel in a previous investigation [4]. Additionally, we evaluated whether knowledge of the government’s role in DS regulation influenced users’ beliefs that DS work and are safe to consume.

## Methods

### Sample population

The survey sample consisted of 990 respondents from 11 military bases – 9 in the U.S. and 2 overseas – and were collected in 2006-7. Survey sites were selected based on the distribution of the Soldier population and their availability. The eligible population included all active-duty U.S Army personnel (a total of 504,422 individuals as of 1 January 2007). Both DS users and nonusers were included in the sample. Survey sites were selected according to the distribution of the soldier population, site availability, and potential to capture a diversity of soldier ranks and job descriptions. Individuals who were on temporary or transitional status, including individuals absent without leave, incarcerated, or moving between permanent duty stations were excluded. Soldiers enrolled in Basic Combat Training or Advanced Individual Training were also excluded, as DS are prohibited during such training. The study was approved by the Institutional Review Board of the United States Army Research Institute of Environmental Medicine (Natick, MA, U.S.A.).

### Survey administration

The data used in this study were obtained from the “Dietary Supplement and Caffeine Intake Survey of US Army Active-Duty Personnel” [4,18]; see Additional file 1 for a copy of the survey. This survey assesses the frequency and reasons for using DS, in addition to demographic and lifestyle information, including questions related to beliefs in the confidence and efficacy of DS. A pilot survey was first conducted with 30 local Army Soldiers to confirm comprehension of study questions and determine time required to complete the survey. Feedback from these volunteers and evaluation of the pilot data indicated volunteers provided reliable and accurate responses to the questions. Following administration of this pilot survey, a contact – typically a dietitian or other health care professional – administered the questionnaire at each study site. The contact arranged with a unit manager or class instructor to distribute the survey at a meeting or class held for another purpose. Typically, when a unit entered the room where the survey was administered, they were seated. A variety of units and classes were approached to ensure representation of all demographic groups. A standardized study briefing was then presented that described the purpose of the survey, which was to assess DS use in the Army. The briefing also described the contents of the survey and its confidential and voluntary nature (no identifying data were collected), and procedures for completing multipart questions. Volunteers then remained in their seats and completed the survey. The completed surveys were returned to the investigators via mail and were scanned and tabulated with ScanTools Plus with ScanFlex (version 6.301; Scantron Corporation, Eagan, MN, U.S.A.), and SPSS (version 15.0; SPSS Inc, Chicago, IL, U.S.A.). Approximately 80% of Soldiers who attended a study briefing opted to complete the survey. Demographic data could not be collected on non-participants, therefore it was not possible to determine whether participants differed from non-participants in any way. However, as 80% of Soldiers given the opportunity to participate did so, and they represented a diverse sample of the Army, response bias is unlikely to have substantially impacted the findings of this study.

### Variables

Two survey questions assessed participants’ confidence in DS: *“How confident are you that your dietary supplements will do as they claim?”* and *“How confident are you that your dietary supplements are safe to consume?”* For each question, participants selected between four response options: *“Extremely confident”, “Very confident”, “Somewhat confident”* or *“Not at all confident”.* Two more questions assessed knowledge of DS regulation: *“Does the U.S. Government require that all dietary supplements sold will work as promised?”* and *“Does the U.S. Government require that all dietary supplements sold are safe for consumption?”* For these two questions, participants answered *“Yes”, “No”,* or *“I don't know”.*

The survey instrument also assessed demographic and lifestyle factors, including sex, age, racial background, military rank, Special Forces status, deployment status, education, military occupation, marital status, tobacco use, aerobic exercise duration, and strength-training participation. Additional questions evaluated usage patterns and reasons for use of both generic supplements (including vitamins, minerals, combination products, antioxidants, herbals, protein and amino acid supplements, and purported steroid analogs) and specific, brand-name products, chosen for inclusion based on then-current patterns of DS purchases at the Army Air Force Exchange System and General Nutrition Center stores on or near Army installations. Participants also had the option to write-in supplements they used that were not listed in the survey. These data are reported elsewhere [4].

### Data analysis

SAS (version 9.2; SAS Institute, Cary, NC, U.S.A.) was used for data analysis. All data were weighted by sex, age, rank, and Special Forces status to represent the overall Army composition as of January 1, 2007. Weights were based on demographic data obtained from the Defense Manpower Data Center (http://www.dmdc.osd.mil/) and the characteristics of survey respondents.

For the purposes of analysis, Soldiers were classified as DS users if they reported consuming a DS (excluding sports drinks, sports bars or gels, and meal replacements) ≥ 1 time/wk during the six months before the survey; all other respondents were classified as nonusers. Standard errors were estimated using a Taylor series linearization method that incorporated sampling weights. Responses to the two high-confidence categories (i.e. “Extremely confident” and “Very confident”) were pooled for analysis.

Category percentages were derived from the ‘surveyfrequency’ procedure in SAS. The ‘surveylogistic’ procedure was used to estimate the likelihood of confidence in DS efficacy or safety (odds ratio and <99% confidence interval) among DS users according to the following participant characteristics: age group; sex; racial background; education; rank; tobacco use; self-rated health, eating habits and fitness level; and reported DS-related adverse events. The likelihood of confidence in DS efficacy and safety was also estimated according to participants’ knowledge of government regulation of DS efficacy and safety. To adjust for multiple comparisons, a Bonferonni adjustment was used for comparisons being made on 26 associations between independent variables and confidence dependent variables. Confidence intervals were calculated with alpha set to 0.0019 in the model statement of the ‘surveylogistic’ procedure. The ‘surveyfrequency’ procedure was also used to derive a Wald chi square test of general association between DS user status and the confidence and knowledge variables. All analyses that required sub-setting the data according to DS user status were performed using the domain statement. We tested for statistical interaction between sex and all predictor variables. However, no interaction term was significant in any model. Thus, all analyses are presented for men and women combined, with the exception of analyses of sex as the independent variable.

## Results

### Sample characteristics

All survey respondents were Active Duty Army personnel. Table 1 displays the characteristics of the sample and frequency of any DS use by demographic group, weighted to represent the full Army composition. Accordingly, demographic percentages were highest for males (86.8%), white/Caucasians (69.5%), 18-24 year olds (41.0%), and enlisted Soldiers (83.5%). More subjects reported their overall health (88.3%), fitness level (76.6%), and eating habits (63.3%) to be “excellent/good” as opposed to “fair/poor”. Most subjects also reported not having experienced an adverse event (86.0%) attributed to DS use. Over half of all respondents (53.2%) used some form of dietary supplement, as defined by DSHEA, at least once per week during the 6 months prior to the survey. Similar to previously published data [4], the percentages of any DS use was highest among those with older age, a Bachelor’s degree or higher, and warrant officers and officers.

**Table 1:** **Characteristics of the study sample**^**1**^

**Characteristic**	**Sample**	**Any DS**^**2**^
	**% (N)**	**% ± SE (N)**
Total	100% (990)	53.2 ± 1.84 (536)
Sex		
Male	86.8 (859)	52.6 ± 1.97 (452)
Female	13.2 (131)	57.3 ± 5.21 (75)
Racial background		
White/Caucasian	69.5 (688)	53.8 ± 2.23 (370)
Black	17.4 (172)	50.8 ± 4.31 (87)
Other	13.2 (130)	53.3 ± 4.98 (69)
Age (years)		
18 to 24	41.0 (406)	41.4 ± 2.58 (168)
25 to 29	21.5 (213)	57.3 ± 3.75 (122)
30 to 39	26.2 (259)	64.0 ± 3.79 (166)
40+	11.3 (112)	63.4 ± 5.88 (112)
Education		
< BS degree	77.5 (767)	48.9 ± 1.92 (375)
≥ BS degree	22.5 (223)	68.0 ± 4.53 (152)
Rank		
Enlisted (1-9)	83.5 (827)	50.5 ± 1.86 (417)
WO/Officer	16.5 (163)	67.2 ± 5.62 (110)
Tobacco use		
Current	43.5 (429)	46.9 ± 2.73 (201)
Former/Never	56.5 (556)	58.1 ± 2.48 (323)
Overall health		
Excellent/Good	88.3 (872)	54.2 ± 1.98 (473)
Fair/Poor	11.7 (115)	45.2 ± 4.92 (52)
Fitness level		
Excellent/Good	76.6 (756)	51.9 ± 3.79 (407)
Fair/Poor	23.4 (232)	53.8 ± 2.12 (120)
Eating habits		
Excellent/Good	63.3 (624)	55.7 ± 2.33 (348)
Fair/Poor	36.7 (362)	49.0 ± 3.01 (177)
Last APFT score		
< 240 or unsure (<median)	29.5 (292)	51.8 ± 2.31
240 - 289 (median)	48.2 (476)	50.3 ± 2.61
≥ 290 (> median)	22.3 (220)	61.3 ± 2.33
Adverse event		
No	86.0 (852)	50.4 ± 1.99 (429)
Yes	14.0 (138)	70.7 ± 4.39 (98)

^1^ Study sample was weighted by sex, age, rank, and Special Forces status to represent the full Army composition as of January, 2007.

^2^ Any DS included all DS at defined by the DSHEA legislation that were reported used at least once a week or more often over the last six months prior to the survey. Any DS excludes sports drinks, sports bars/gels and meal replacement beverages.

In addition, proportionately more former or never smokers (58.1%) used DS compared to current smokers (46.9%), as well as those that rated their overall health and eating habits as excellent/good compared to fair/poor (54.2% vs. 45.2% and 55.7% vs. 49%, respectively). Proportionately more subjects with APFT scores above the median category (61.3%) used DS than those with APFT scores at the median (50.3%) or below (51.8%). The majority of subjects reported not experiencing an adverse event (86.0%).

### Confidence in DS efficacy

**Figure 1:**
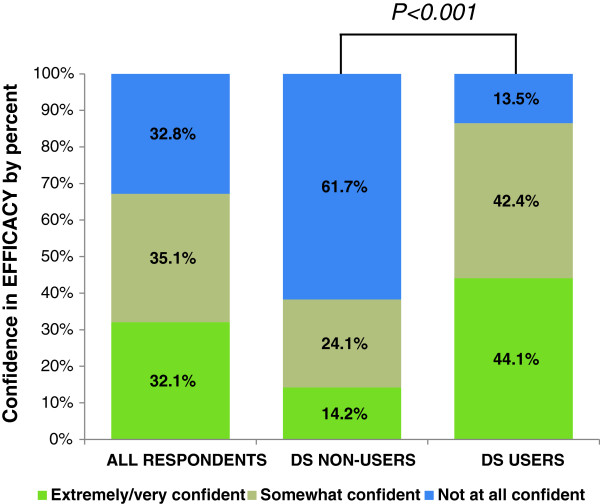
**Percentage of respondents reporting degree of confidence in DS efficacy (extremely/very confident, somewhat confident, or not at all confident) for all respondents and according to DS user status.** P < 0.001 indicates a significant association between DS use status and confidence according to the Wald chi-square test.

Two-thirds (67%) of all respondents were at least somewhat confident that DS work as advertised, and approximately half had high confidence (i.e. selected “Extremely” or “Very confident”) (Figure 1). Confidence in the purported efficacy of DS differed significantly by user status (p < .001); 86.5% of users had at least some confidence that DS work as advertised, compared to only 38.3% of non-users. Most non-users (61.7%) were “Not at all confident” that DS work as advertised; however, 13.5% of the respondents who used DS also endorsed this response option. Among users, beliefs regarding DS efficacy did not significantly differ significantly by age, sex, racial background, education, or rank (Table 2).

**Table 2:** Likelihood of confidence in efficacy of DS among DS users according to demographics, lifestyle, and health characteristics

	**Extremely/Very confident**		**Somewhat confident**		**Not at all confident**	
	**% (N)**	**OR (<99% CI)**	**% (N)**	**OR (<99% CI)**	**% (N)**	**OR (<99% CI)**
Sex						
Female (65)	45.1 (29)	1.00	38.4 (25)	1.00	16.4 (11)	1.00
Male (422)	43.9 (185)	0.95 (0.35, 2.60)	43.0 (181)	1.21 (0.44, 3.34)	13.1 (55)	0.77 (0.22, 2.72)
Racial background						
Caucasian or white (345)	45.2 (156)	1.00	41.4 (143)	1.00	13.4 (46)	1.00
African American or black (77)	36.0 (28)	0.68 (0.28, 1.69)	48.9 (37)	1.35 (0.55, 3.31)	15.1 (12)	1.16 (0.36, 3.75)
Other (64)	47.6 (31)	1.10 (0.41, 2.95)	39.9 (26)	0.94 (0.35, 2.51)	12.5 (8)	0.92 (0.23, 3.72)
Age						
18-24 (152)	42.0 (64)	1.00	41.2 (63)	1.00	16.8 (26)	1.00
25-29 (116)	39.7 (46)	0.91 (0.39, 2.11)	44.0 (51)	1.13 (0.49, 2.63)	16.1 (19)	0.95 (0.31, 2.93)
30-39 (149)	51.4 (76)	1.46 (0.62, 3.45)	38.7 (58)	0.90 (0.38, 2.17)	9.9 (15)	0.54 (0.16, 1.80)
40+ (69)	40.4 (28)	0.91 (0.39, 2.11)	49.8 (34)	1.41 (0.48, 4.17)	9.9 (7)	0.54 (0.14, 2.14)
Education						
< Bachelor degree (352)	43.2 (152)	1.00	40.6 (143)	1.00	16.2 (57)	1.00
≥ Bachelor degree (134)	46.5 (62)	1.14 (0.49, 2.68)	47.0 (63)	1.30 (0.55, 3.03)	6.5 (9)	0.36 (0.11, 1.25)
Rank						
Enlisted (393)	43.2 (170)	1.00	40.6 (162)	1.00	16.2 (61)	1.00
Officer (93)	47.4 (44)	1.18 (0.41, 3.40)	47.5 (44)	1.30 (0.45, 3.72)	5.1 (5)	0.29 (0.07, 1.26)
Tobacco Use						
Current (193)	41.4 (80)	1.00	44.9 (87)	1.00	13.7 (26)	1.00
Former or never (291)	46.0 (134)	1.20 (0.60, 2.40)	40.5 (118)	0.84 (0.42, 1.67)	13.5 (39)	0.99 (0.40, 2.45)
Overall health						
Fair/Poor (50)	31.8 (16)	1.00	48.5 (24)	1.00	19.7 (10)	1.00
Excellent/Good (435)	45.7 (199)	1.80 (0.62, 5.29)	41.4 (180)	0.75 (0.27, 2.07)	12.9 (56)	0.60 (0.17, 2.09)
Fitness level						
Fair/Poor (114)	28.7 (33)	1.00	50.5 (57)	1.00	20.8 (24)	1.00
Excellent/Good (373)	48.8 (182)	2.37 (1.01, 5.57)	39.9 (149)	0.65 (0.30, 1.43)	11.3 (42)	0.48 (0.19, 1.27)
Eating habits						
Fair/Poor (170)	30.4 (52)	1.00	53.5 (91)	1.00	16.1 (27)	1.00
Excellent/Good (314)	51.8 (163)	2.47 (1.19, 5.11)	36.0 (113)	0.49 (0.24, 0.99)	12.2 (38)	0.73 (0.29, 1.82)
Ever enrolled in AWCP						
Yes (53)	36.0 (19)	1.00	50.6 (27)	1.00	13.4 (7)	1.00
No (429)	45.3 (194)	1.47 (0.48, 4.49)	41.0 (176)	0.68 (0.23, 2.02)	13.7 (59)	1.03 (0.25, 4.19)
Last APFT score						
< 240 or unsure (138)	35.5 (49)	1.00	42.6 (59)	1.00	21.8 (30)	1.00
≥ 240 (346)	47.7 (165)	1.66 (0.78, 3.51)	42.0(145)	0.97 (0.47, 2.02)	10.3 (36)	0.41 (0.17, 1.03)
Adverse event						
No (391)	47.1 (184)	1.00	38.3 (150)	1.00	14.7 (57)	1.00
Yes (95)	31.7 (30)	0.52 (0.22, 1.22)	59.3 (56)	2.35 (1.03, 5.38)	9.00 (9)	0.57 (0.16, 2.02)

Among DS users, self-reported fitness level and eating habits were both significantly associated with beliefs about DS efficacy (Table 2). Those who reported fitness levels as “Excellent/Good” were more than twice as likely to be highly confident in DS efficacy than those who reported fitness levels as “Fair/Poor” (OR = 2.37, CI = 1.01-5.57). Those who reported their eating habits as “Excellent/Good” were nearly two and half times as likely to be extremely/very confident (OR = 2.47, CI = 1.19-5.11) and approximately 50% less likely to be somewhat confident (OR = 0.49, CI = 0.24-0.99) in DS efficacy than those who reported their eating habits as “Fair/Poor”. On the other hand, neither perceived overall health status, nor tobacco use (current vs. former/never) was related to confidence in DS efficacy. Finally, a significant association was observed between self-reported adverse events and confidence, such that participants who believed they had experienced an adverse event due to DS usage were over two times more likely to be somewhat confident in DS efficacy than those who did not experience an adverse event (OR = 2.35, CI = 1.03-5.38), but were less likely to be either extremely/very confident or not at all confident, although these associations were not significant.

### Confidence in DS safety

**Figure 2:**
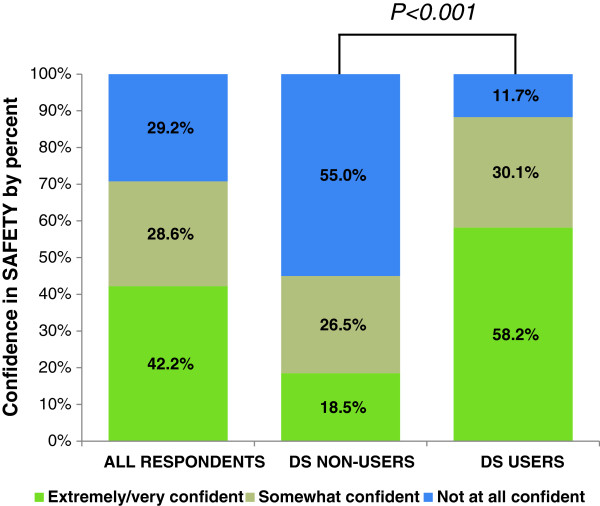
**Percentage of respondents reporting degree of confidence in DS safety (extremely/very confident, somewhat confident, or not at all confident) for all respondents and according to DS user status.** P < 0.001 indicates a significant association between DS use status and confidence according to the Wald chi-square test.

Soldiers had slightly more confidence in DS safety than efficacy; 70.8% of all respondents were at least somewhat confident DS are safe to consume, and of them, 42.2% reported high confidence (Figure 2). Confidence in the safety of DS differed significantly by user status (p < .001). Eighty-eight percent of users reported at least some confidence in DS safety, while less than half (45.0%) of non-users reported at least some confidence. Surprisingly, while 55.0% of non-users were “Not at all confident” that DS are safe to consume, 11.7% of users also indicated they had no confidence in DS safety.

Among users, reported confidence in DS safety did not differ by sex or racial background (Table 3). In general, all older age groups were more likely to be somewhat confident and less likely to be not at all confident in DS safety than 18-24 year olds, but these associations were only significant for 25-29 year olds (somewhat confident; OR = 2.06, CI = 1.16-3.65) and 30-39 year olds (not at all confident; OR = 0.38, CI = 0.17, 0.84). Both education status and rank were also associated with how participants viewed the safety of their supplements; Participants who had completed a bachelor’s degree or higher and officers were less likely to be not at all confident in DS safety than those with less education (OR = 0.41, CI = 0.19-0.93) or enlisted personnel (OR = 0.30, CI = 0.11-0.83), respectively. Former or individuals who had never smoked were less likely to be somewhat confident in DS safety than current smokers (OR = 0.61, CI = 0.38-0.96). Interestingly, former or individuals who had never smoked were more likely to be both extremely/very confident and not at all confident, but these associations were not significant.

**Table 3:** Likelihood of confidence in safety of DS among DS users according to demographics, lifestyle, and health characteristics

	**Extremely/Very confident**		**Somewhat confident**		**Not at all confident**	
	**% (N)**	**OR (<99% CI)**	**% (N)**	**OR (<99% CI)**	**% (N)**	**OR (<99% CI)**
Sex						
Female (66)	52.2 (35)	1.00	33.5 (22)	1.00	14.2 (19)	1.00
Male (422)	59.1 (249)	1.32 (0.50, 3.54)	29.5 (125)	0.83 (0.43, 1.60)	11.3 (48)	0.77 (0.33, 1.78)
Racial background						
Caucasian or white (345)	59.3 (205)	1.00	30.1 (104)	1.00	10.7 (37)	1.00
African American or black (77)	53.6 (41)	0.80 (0.33, 1.93)	32.9 (25)	1.14 (0.64, 2.05)	13.4 (10)	1.30 (0.60, 2.83)
Other (66)	57.8 (38)	0.94 (0.36, 2.48)	26.7 (18)	0.85 (0.44, 1.64)	15.4 (10)	1.53 (0.67, 3.47)
Age						
18-24 (153)	58.9 (90)	1.00	23.7 (36)	1.00	17.4 (27)	1.00
25-29 (118)	49.9 (59)	0.70 (0.30, 1.61)	39.0 (46)	2.06 (1.16, 3.65)	11.1 (13)	0.59 (0.27, 1.29)
30-39 (149)	61.4 (91)	1.11 (0.47, 2.62)	31.2 (46)	1.46 (0.81, 2.63)	7.4 (11)	0.38 (0.17, 0.84)
40+ (69)	64.0 (44)	1.25 (0.43, 2.63)	26.3 (18)	1.15 (0.54, 2.43)	9.7 (7)	0.51 (0.21, 1.22)
Education						
< Bachelor degree (354)	54.3 (192)	1.00	31.9 (113)	1.00	13.8 (49)	1.00
≥ Bachelor degree (134)	68.6 (92)	1.84 (0.78, 4.36)	25.2 (34)	0.72 (0.40, 1.29)	6.2 (8)	0.41 (0.19, 0.93)
Rank						
Enlisted (395)	54.5 (215)	1.00	32.1 (127)	1.00	13.4 (53)	1.00
Officer (93)	74.0 (69)	2.38 (0.79, 7.21)	21.5 (20)	0.58 (0.27, 1.24)	4.5 (4)	0.30 (0.11, 0.83)
Tobacco Use						
Current (194)	53.4 (103)	1.00	36.3 (70)	1.00	10.3 (20)	1.00
Former or never (292)	61.5 (179)	1.39 (0.71, 2.75)	25.6 (75)	0.61 (0.38, 0.96)	12.8 (38)	1.29 (0.70, 2.38)
Overall health						
Fair / Poor (51)	52.5 (27)	1.00	33.1 (17)	1.00	14.4 (7)	1.00
Excellent / Good (435)	58.9 (256)	1.30 (0.48, 3.53)	29.6 (129)	0.85 (0.43, 1.68)	11.5 (50)	0.77 (0.33, 1.79)
Fitness level						
Fair / Poor (115)	44.1 (51)	1.00	40.0 (46)	1.00	15.9 (18)	1.00
Excellent / Good (373)	62.6 (233)	2.12 (0.95, 4.71)	27.0 (101)	0.56 (0.34, 0.92)	10.4 (39)	0.62 (0.32, 1.18)
Eating habits						
Fair / Poor (173)	46.0 (80)	1.00	40.0 (69)	1.00	14.0 (24)	1.00
Excellent / Good (313)	65.0 (203)	2.18 (1.07, 4.43)	24.5 (77)	0.49 (0.31, 0.77)	10.5 (33)	0.72 (0.39, 1.33)
Ever enrolled in AWCP						
Yes (53)	50.4 (27)	1.00	38.0 (20)	1.00	11.6 (6)	1.00
No (431)	59.2 (255)	1.43 (0.48, 4.23)	28.9 (125)	0.66 (0.34, 1.32)	11.9 (51)	1.03 (0.40, 2.64)
Last APFT score						
< 240or unsure (136)	44.9 (62)	1.00	37.5 (52)	1.00	17.7 (24)	1.00
≥ 240 (367)	63.5 (221)	2.14 (1.03, 4.46)	27.0 (94)	0.62 (0.39, 0.99)	9.4 (33)	0.49 (0.26, 0.89)
Adverse event						
No (393)	63.4 (249)	1.00	24.7 (97)	1.00	11.9 (47)	1.00
Yes (95)	36.8 (35)	0.34 (0.14, 0.79)	52.2 (50)	3.33 (1.95, 5.70)	11.0 (10)	0.92 (0.44, 1.93)

There were significant relationships between both self-reported fitness-level and eating habits and participants’ confidence in DS safety. Those who reported their fitness level and eating habits as “excellent/good” were less likely to be somewhat confident in safety than those who reported those factors as “fair/poor” (OR = 0.56, CI = 0.34-0.92 and OR = 0.49, CI = 0.31-0.77, respectively). Those who reported their eating habits as “excellent/good” were also over twice as likely to be extremely/very confident in safety than those who reported their habits as “fair/poor” (OR = 2.18, CI = 1.07-4.43). Conversely, neither self-reported overall health status nor enrollment in the Army Weight Control Program were related to participants’ beliefs that supplements are safe to consume. Those who reported scoring at or above the median category of Army Physical Fitness Test (APFT) score (≥ 240) were over two times more likely to be extremely/very confident in safety (OR = 2.14, CI = 1.03, 4.46) and less likely to be somewhat and not at all confident in safety (OR = 0.62, CI = 0.39, 0.99 and OR = 0.49, CI = 0.26-0.89, respectively). Users who believed they had experienced one or more adverse events from taking DS were less likely to be extremely/very confident in safety (OR = 0.34, CI = 0.14-0.79) and more likely to be only somewhat confident in safety (OR = 3.33, CI = 1.95-5.7) than those who did not report experiencing an adverse event.

### Knowledge of government regulation

**Figure 3:**
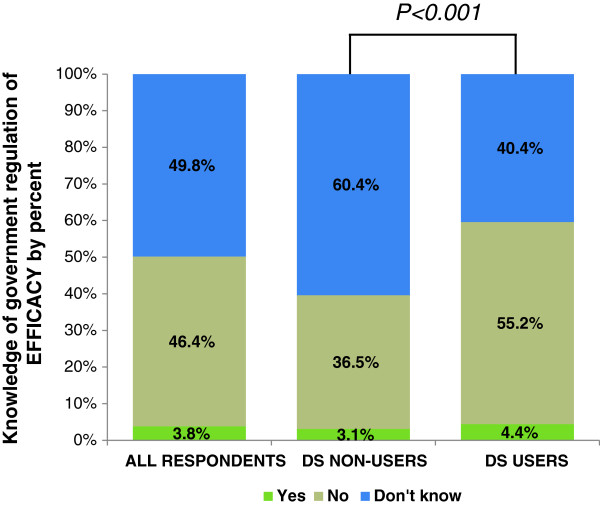
**Percentage of respondents reporting knowledge of government regulation of DS efficacy for all respondents and according to DS user status.** Respondents prompted to answer ‘Yes’, ‘No’, or ‘Don’t know’ to the following question, “Does the U.S. Government require that all dietary supplements sold will work as promised?” P < 0.001 indicates a significant association between DS use status and knowledge of government regulation of DS efficacy according to the Wald chi-square test.

**Figure 4:**
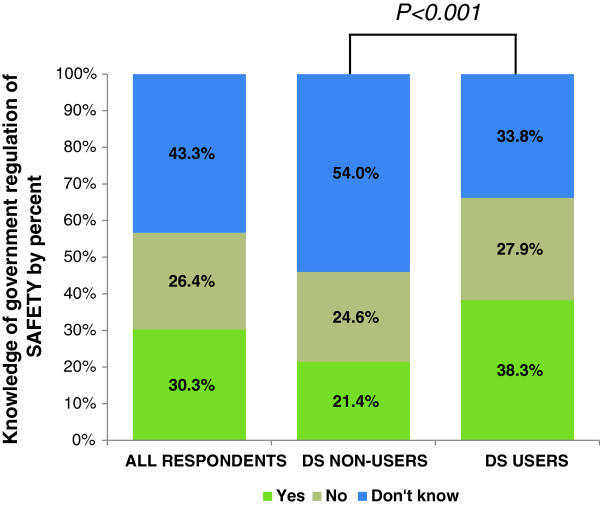
**Percentage of respondents reporting knowledge of government regulation of DS safety for all respondents and according to DS user status.** Respondents prompted to answer ‘Yes’, ‘No’, or ‘Don’t know’ to the following question, “Does the U.S. Government require that all dietary supplements sold are safe for consumption?” P < 0.001 indicates a significant association between DS use status and knowledge of government regulation of DS safety according to the Wald chi-square test.

Nearly half of all respondents did not know whether the U.S. government requires DS to work as advertised and 46.4% knew it does not. Only 3.8% of all respondents incorrectly believed the government requires DS to be effective (Figure 3). However, many Soldiers (30.3%) believed the government requires all marketed DS to be safe for consumption (Figure 4).

Knowledge about DS regulation for efficacy and safety differed significantly by user status (p < .001; p < .001) (Figures 3 and 4). Most users (55.2%) knew the government does not require DS to work as advertised, whereas most non-users (60.4%) did not know whether or not there are federal regulations for DS efficacy. Also, proportionately more users than non-users believed that the U.S. Government requires DS to be safe for consumption (38.3% and 21.4%, respectively). Beliefs about federal DS regulation were significantly associated with how confident users were in the efficacy and safety of supplements (Table 4). Users who believed the government does not require all DS sold to be effective were less likely to be extremely/very confident in DS efficacy compared to those who did believe (OR = 0.40, CI = 0.11-0.99). Users who did not know whether the government requires all DS sold to be safe were less likely to be extremely/very confident (OR = 0.43, CI = 0.26-0.71) and more likely to be not at all confident (OR = 6.52, CI = 2.58-16.5) in DS safety than those who did believe the government requires all DS sold to be safe. Those who did not believe the government requires all DS sold to be safe were also more likely to be not at all confident in DS safety than those who did believe (OR = 3.94, CI = 1.47-10.5).

**Table 4:** Likelihood of confidence in efficacy and safety of DS according to knowledge of federal government regulation Does the U.S. government require dietary supplements sold will work as promised?

	**Confidence in DS efficacy**					
	**Extremely/Very confident**		**Somewhat confident**		**Not at all confident**	
	**% (N)**	**OR (<99% CI)**	**% (N)**	**OR (<99% CI)**	**% (N)**	**OR (<99% CI)**
Yes (20)	66.9 (14)	1.00	28.8 (6)	1.00	4.3 (1)	1.00
No (269)	44.8 (121)	0.40 (0.11, 0.99)	44.7 (120)	2.00 (0.66, 6.08)	10.5 (28)	2.60 (0.33, 20.9)
Don’t know (187)	39.9 (74)	0.33 (0.14, 1.19)	40.9 (76)	1.72 (0.56, 5.30)	19.2 (36)	5.25 (0.65, 42.1)
Does the U.S. government require dietary supplements sold are safe to consume?						
	**Confidence in DS safety**					
	**Extremely/Very confident**		**Somewhat confident**		**Not at all confident**	
	**% (N)**	**OR (<99% CI)**	**% (N)**	**OR (<99% CI)**	**% (N)**	**OR (<99% CI)**
Yes (177)	68.5 (121)	1.00	27.8 (49)	1.00	3.6 (6)	1.00
No (134)	58.6 (79)	0.65 (0.38, 1.12)	28.5 (38)	1.03 (0.57, 1.86)	12.9 (17)	3.94 (1.47, 10.5)
Don’t know (169)	48.2 (82)	0.43 (0.26, 0.71)	32.1 (54)	1.23 (0.72, 2.08)	19.7 (33)	6.53 (2.58, 16.5)

## Discussion

This study is the first to assess beliefs about DS efficacy and safety among U.S. Army Soldiers and to examine demographic and lifestyle factors are associated with higher confidence in DS efficacy or safety among DS users. Additionally, it is the first to assess whether knowledge of federal DS regulatory requirements affected users’ perceptions of supplement efficacy or safety. We found most Soldiers were at least somewhat confident DS work as advertised and are safe to consume, that confidence in DS efficacy and safety was higher among users compared to non-users, and that users who had not experienced DS-related adverse events had higher confidence in both attributes. Most Soldiers had limited or inaccurate knowledge of federal DS regulatory requirements. Furthermore, confidence in DS efficacy and safety was higher among users who believed government regulations require that all marketed supplements work as advertised and are safe to use.

### Confidence in DS efficacy and safety

Confidence in both DS efficacy and safety was substantially greater among regular supplement users, who make up about half the Army population. The positive association between usage and confidence in DS is not surprising, and is consistent with other reports. For example, Blendon et al. [2] showed regular DS users, compared to non-users, were more likely to believe that advertisements about DS are generally true, that DS undergo adequate pre-market testing, and that DS “rarely or never” harm the user. Likewise, individuals who use herbal supplements or OTC weight-loss aids were found to be more likely to perceive such products as effective and/or safe compared to nonusers [16,17]. Marketing research has shown that direct product exposure (e.g. sampling or using a product) results in higher, and more firmly-held beliefs and attitudinal confidence in the product compared to indirect product exposure (e.g. viewing advertising materials) [19], presumably because people generally trust their own judgment, but recognize that advertisements are often biased. Thus, DS users may be more likely to believe DS work and are safe, simply because they have tried the product, even if the product is ineffective. It is also possible that individuals with low confidence in DS are less likely to begin using the product in the first place.

Our findings indicate DS users’ product confidence was positively related to self-rated diet quality, perceived fitness level, and rank. These associations may be due in part to participants’ level of optimism and/or self -confidence. Individuals with healthier lifestyles are more likely to have an optimistic cognitive bias compared to those with less healthy behaviors [20], and thus may be predisposed to believe DS are efficacious and safe. Similarly, Soldier rank correlates positively with self-confidence [21], so officers may be more likely to believe their actions are purposeful and beneficial (e.g. that consuming DS is efficacious and safe) compared to enlisted personnel. Because U.S. Army officers generally have more formal education compared to enlisted personnel, our observation that DS confidence in safety increased with education may be a reflection of respondents’ rank and, hence, self-confidence. This may explain why our result differs from other studies, which reported lesser-educated individuals more likely to believe DS are effective and/or safe [7,9].

On the whole, these observations suggest that DS users’ confidence in product efficacy and safety is partly dependent on internal factors – such as self-confidence and optimism. Self-confident individuals may not seek out accurate product knowledge (i.e. from scientific sources) because they trust their ability to evaluate the veracity of product information, regardless of source; and optimistic individuals may not seek out scientific confirmation because they are already inclined to believe the product will work.

### Knowledge of DS regulation

Most Soldiers had a limited or inaccurate understanding of the U.S. government’s role in regulating DS, which reflects what has been reported in the general American population [2,7,13,14]. Of note, Soldiers were more apt to believe the government requires DS to be safe than to think the government imposes strict efficacy requirements, particularly if they used DS. Twice as many users as non-users incorrectly believed the U.S. government requires DS to be safe. The reasons for this difference are not clear. Confidence may be influenced by the disclaimer statement required on many DS, which states that the FDA has not approved any health or structure-function efficacy claims made on the label, but says nothing about product safety. Consumers may interpret this *lack* of a safety disclaimer to mean the product is not harmful (see Dodge and Kaufman, 2007 [15]). One limitation of this study is that we did not separately assess participants’ confidence in each supplement, or for each type of supplement (e.g. protein/amino-acid supplements, vitamins/minerals, etc.), therefore, it is not possible to conclude whether users’ confidence in DS varies between individual types of supplements. Future research should investigate whether confidence varies by supplement type. Additionally, Soldiers may have misinterpreted the questions about government regulation of DS, since even without pre-market approval requirements for efficacy and safety, there is some limited *de facto* regulation by the U.S. government in the form of post-market FDA surveillance.

Information on Soldiers’ level of confidence in DS may be useful when developing educational strategies for Soldiers about DS, as confidence and beliefs can affect how people receive information on a particular topic. In addition, these strategies should also consider that DS users may already engage in health behaviors. For example, self-reported health behaviors, including smoking, overall health, and fitness levels appeared to be related to DS use in this study. Those in the highest APFT score category also reported the highest percentage of DS use. While the majority of subjects have not experienced an adverse event (86.0%), 14% did report experiencing an event, indicating that although adverse events are not widespread, they do occur. Taken together, these data suggest that the behavioral interventions aimed at motivated individuals who use DS in conjunction with health behaviors may best be focused on providing education on evaluating DS efficacy and safety. This may aid individuals in making decisions regarding DS use to optimize effectiveness, while minimizing the risk of experiencing an adverse event. Results of this study indicate that education to clarify Soldiers’ misperceptions about federal DS safety and efficacy regulations is warranted. Furthermore, because an individual’s beliefs regarding the value of a particular action (i.e. use of a particular supplement) may influence his/her his motivation to change that behavior [22], an intervention approach that works in individuals with low confidence will likely not be effective in highly-confident users. In this survey, respondents most frequently cited magazines, friends, and the internet (data not shown) as the source of their dietary supplement information, thus these sources may be a potential target of educational interventions.

## Conclusions

This study expands the existing literature on dietary supplements by exploring factors associated with DS users’ confidence in these products, and is the first to investigate beliefs regarding efficacy and safety of DS in a military population. Although there is limited scientific evidence in support of manufactures’ claims regarding the benefits and safety of most popular DS, this analysis demonstrated most Soldiers were at least somewhat confident that these products are effective and safe. In general, confidence in both attributes was higher among users compared to non-users, and among users, is positively associated with rank, education, self-perceived diet quality and fitness level, and having experienced no adverse events resulting from DS consumption. The positive associations between confidence and DS use should be considered when developing DS-related interventions or policies. Education to clarify Soldiers’ misperceptions about federal DS safety and efficacy regulations is warranted. Additionally, future studies should consider surveying a matched group of civilians for comparison to Soldiers*.*

## Abbreviations

APFT: Army Physical Fitness Test; DS: Dietary Supplement; FDA: Food and Drug Administration; DSHEA: Dietary Supplement Health and Education Act.

## Competing interests

The authors declare that they have no competing interests.

## Authors’ contributions

CEC participated in the design of the study, performed preliminary statistical analyses, and drafted the manuscript. EF conducted the final statistical analyses, and contributed to data interpretation and manuscript revision. HRL conceived the study design and helped draft the manuscript. All authors read and approved the final manuscript.

## Pre-publication history

The pre-publication history for this paper can be accessed here:

http://www.biomedcentral.com/1472-6882/12/182/prepub

## Supplementary Material

Additional file 1 Dietary Supplement Survey (E06-21). Click here for file
