# Supplementary material for: Confidence in the efficacy and safety of dietary supplements among United States active duty army personnel
Source: BMC Complement Altern Med. 2012 Oct 10;12:182. doi: 10.1186/1472-6882-12-182 (PMC3598849; doi:10.1186/1472-6882-12-182)
Supplement: Additional file 1 — Dietary Supplement Survey (E06-21). [file 1472-6882-12-182-S1.pdf]

Dietary Supplement Survey (E06-21)

This study is completely anonymous, participating is voluntary and you are free to withdraw from the study

This questionnaire asks a variety of questions about your background, eating and exercise habits, how you are feeling, and dietary supplement use to assess dietary supplement use in the Army. This questionnaire is strictly voluntary and your answers will be kept confidential. This will take about 25 minutes of your time. Thank you for your participation.

MARKING INSTRUCTIONS
• Use a No. 2 pencil only.<br>• Do not use ink, ballpoint, or felt tip pens.<br>• Make solid marks that fill the response completely.<br>• Erase cleanly any marks you wish to change.<br>• Make no stray marks on this form.<br>CORRECT: ● INCORRECT: ✓ ☒ ◒ ◔

Below you will find an example of a question from this booklet. Please note the proper way to record your responses.

Example:

How long have you been in the Armed Services?
If your answer is 5 years, then you would write the numbers 0 and 5 in the boxes and then darken the corresponding circles. Please make sure that you use leading zeros when needed.

Please write in your response in the blank boxes, then fill in the corresponding circles.

years	0	● 1 2 3 4 5 6 7 8 9
	5	0 1 2 3 4 ● 6 7 8 9

U.S. ARMY RESEARCH INSTITUTE OF ENVIRONMENTAL MEDICINE (USARIEM)
MILITARY NUTRITION DIVISION
NATICK, MA 01760

PLEASE DO NOT WRITE IN THIS AREA

[SERIAL]

Dietary Supplement Survey

BACKGROUND QUESTIONS

1. Gender:

- (M) Male
- (F) Female

2. What is your age today?

AGE years	
0	0
1	1
2	2
3	3
4	4
5	5
6	6
7	7
8	8
9	9

3. Your height in inches?
(without shoes/boots)

HEIGHT inches	
0	0
1	1
2	2
3	3
4	4
5	5
6	6
7	7
8	8
9	9

4. Your weight in pounds?
(without clothing)

WEIGHT pounds		
0	0	0
1	1	1
2	2	2
3	3	3
4	4	4
5	5	5
6	6	6
7	7	7
8	8	8
9	9	9

5a. What is your ethnic background?

- (1) Hispanic or Latino
- (2) Not Hispanic or Latino

5b. What is your racial background?

- (1) White or Caucasian
- (2) Black or African American
- (3) Native American/Alaskan Native
- (4) Asian
- (5) Native Hawaiian/Pacific Islander
- (6) Other__________________

Do not write in this box
0 1 2 3 4 5 6 7 8 9
0 1 2 3 4 5 6 7 8 9
Q5b_oth

6. What is your rank?

E	1 2 3 4 5 6 7 8 9
O	1 2 3 4 5 6 7 8 9
WO	1 2 3 4 5 6

7. What is your current area of assignment?

- (1) Combat arms (Infantry, Armor, Field Artillery, Air Defense, Special Forces)
- (2) Combat support (Engineer, Chemical, Military Intelligence, Military Police, Signal, Aviation, Civil Affairs)
- (3) Combat service support (Ordnance, Quartermaster, Transportation, Adjutant, Medical, Finance, Chaplain, Judge advocate General, Inspector General)

8. What is your primary MOS? Description:_________________________________

	0 1 2 3 4 5 6 7 8 9
	0 1 2 3 4 5 6 7 8 9
	A B C D E F G H I J K L M N O P Q R S T U V W X Y Z

9. Please indicate the highest level of education you have completed.

- (1) Some high school (but no GED or diploma)
- (2) High school graduate (GED or diploma)
- (3) Some college courses
- (4) Associate degree (two-year college)
- (5) Bachelors degree (four-year college)
- (6) Graduate degree

10. What is your marital status? Please fill in one circle.

- ① Single, never married
- ② Married
- ③ Not married (Widowed/Divorced)

11. Where do you live now?

- ① Barracks
- ② Post family housing
- ③ B.O.Q.
- ④ Off-post housing

12. How long have you been in the Armed Services?

Active

① Less than one year **OR** If a year or more, please fill in the number of years (start with leading zero's when needed).

0 1 2 3 4 5
0 1 2 3 4 5 6 7 8 9

13. How many times have you been deployed to a combat zone, in your military career? If none, fill in 00 and go to question #16.

number of tours

0 1 2 3 4 5 6 7 8 9
0 1 2 3 4 5 6 7 8 9

⓪ Not sure

14. What is your total combined time deployed to a combat zone, in years and months?

Years

0 1 2 3 4 5 6 7 8 9
0 1 2 3 4 5 6 7 8 9

Months

0 1 2 3 4 5 6 7 8 9
0 1 2 3 4 5 6 7 8 9

⓪ Not sure

15. When did you last return from a deployment to a combat zone?

Month

0 1
0 1 2 3 4 5 6 7 8 9

Year

0 1 2 3 4 5 6 7 8 9
0 1 2 3 4 5 6 7 8 9
0 1 2 3 4 5 6 7 8 9
0 1 2 3 4 5 6 7 8 9

⓪ Not sure

16. Are you scheduled for deployment to a combat zone in the next 6 months?

- ① Yes
- ② No
- ③ Not sure

Dietary Supplement Survey

HEALTH QUESTIONS

17. How do you consider your general health?

1 Excellent
2 Good
3 Fair
4 Poor

18. How do you consider your overall eating habits?

1 Excellent
2 Good
3 Fair
4 Poor

19. Which of the following are you currently trying to do about your weight? Please circle only ONE response

1 Trying to **LOSE** weight
2 Trying to **GAIN** weight
3 Maintaining weight

20. Have you ever been enrolled in the Army Weight Control Program?

1 Yes
2 No
3 Not sure

21. What did you score on your last APFT?

1 ≥ 300
2 290 - 299
3 240 - 289
4 180 - 239
5 < 180
6 Not sure, passed
7 Not sure, failed

22. Which best describes your diet? *(Please mark all that apply)*

1 Weight loss
2 Vegetarian/Vegan
3 Low salt/sodium
4 Weight gain
5 Cholesterol lowering
6 High Protein
7 Low Fat
8 High Carbohydrate
9 Low Carbohydrate
No special diet
Other, please specify:____

0 1 2 3 4 5 6 7 8 9
0 1 2 3 4 5 6 7 8 9
0 1 2 3 4 5 6 7 8 9
oth_diet

23. Which statement best describes your use of tobacco products (cigarettes, pipes, cigars, smokeless tobacco - chew, dipping, pinching) in the <u>last year</u>?

1. I have never used tobacco products
2. I used tobacco products but quit
3. I use tobacco products 3 or less times per week
4. I use tobacco products 4 - 6 times per week
5. I use tobacco products at least one time per day

EXERCISE QUESTIONS

24. How do you consider your overall fitness level?

1. Excellent
2. Good
3. Fair
4. Poor

25. Aerobic exercise includes activities such as running, cycling, stair climbing, swimming, and road marching. Use the table below to describe the frequency and duration of your nonstop aerobic exercise with your unit and on your own time. You may choose more than one duration with your unit and/or on your own time.

<u>Exercise Frequency</u>:

<u>Exercise Duration</u>:	RARELY/NEVER	1 DAY PER WEEK	2 DAYS PER WEEK	3 DAYS PER WEEK	4 DAYS PER WEEK	5 DAYS PER WEEK	6 DAYS PER WEEK	7 DAYS PER WEEK	MULTIPLE DAILY SESSIONS
**WITHIN THE UNIT**									
< 30 minutes	0	1	2	3	4	5	6	7	8
30 - 60 minutes	0	1	2	3	4	5	6	7	8
61 - 90 minutes	0	1	2	3	4	5	6	7	8
> 90 minutes	0	1	2	3	4	5	6	7	8
**ON OWN TIME**									
< 30 minutes	0	1	2	3	4	5	6	7	8
30 - 60 minutes	0	1	2	3	4	5	6	7	8
61 - 90 minutes	0	1	2	3	4	5	6	7	8
> 90 minutes	0	1	2	3	4	5	6	7	8

26. How often do you lift weights or participate in other forms of strength conditioning exercise? Use the table below to describe the frequency of your strength conditioning exercise with your unit and on your own time.

**WITHIN THE UNIT**	0	1	2	3	4	5	6	7	8
**ON OWN TIME**	0	1	2	3	4	5	6	7	8

27. Why do you exercise? (choose all that apply)

1. Health reasons
2. Increase muscle mass
3. Preparing for a strength competition
4. Preparing for an aerobic competition
5. Fun
6. Stress relief
7. To prepare for the APFT
8. I don't exercise
9. Weight loss
10. Physician directed
11. Other__________________

Do not write in this box
0 1 2 3 4 5 6 7 8 9
0 1 2 3 4 5 6 7 8 9
Q41_oth

28. Complete the following by filling in the appropriate circle for each adjective, to give an indication of your mood at this moment. Please work quickly: do not take too long thinking about each answer.

	NOT AT ALL	A LITTLE	MODERATELY	VERY	EXTREMELY
Wide awake	0	1	2	3	4
Relaxed	0	1	2	3	4
Depressed	0	1	2	3	4
Friendly	0	1	2	3	4
Anxious	0	1	2	3	4
Clumsy	0	1	2	3	4
Cheerful	0	1	2	3	4
Drowsy	0	1	2	3	4
Agressive	0	1	2	3	4
Clear-headed	0	1	2	3	4
Well-coordinated	0	1	2	3	4
Confused	0	1	2	3	4

29. Do you know the ingredients contained in your dietary supplements?

1. All the ingredients
2. Most of the ingredients
3. Some of the ingredients
4. None of the ingredients
5. I do not take supplements

30. Do any of the supplements you use contain caffeine?

1. Yes
2. No
3. Not sure

31. Does the U.S. Government require that all dietary supplements sold will work as promised?

1. Yes
2. No
3. I don't know

32. Does the U.S. Government require that all dietary supplements sold are safe for consumption?

1. Yes
2. No
3. I don't know

Dietary Supplement Survey

33. CAFFEINE INSTRUCTIONS:

1) Indicate your serving size for each item you consume
2) Fill in a bubble for the number of times you usually consume that item. If you do not consume that item, fill in "0" under TIMES column, leaving all other sections blank.
3) Fill in the bubble indicating the time period for the number of times you usually have that item in that amount.

For sodas: a can = 12 fl oz, vending plastic bottles = 20 fl oz, and a 2-liter bottle is 68 fl oz).
Example: A person drinks three 8-fl oz cups of coffee, 2 times a day.

Item	8 fl oz / 12 fl oz / 16 fl oz / 20 fl oz / 24+ fl oz	--- Times ---	Day / Week / Month / Year	Do not write in this box
**COFFEE/TEA (caffeinated only)**				0 0
EXAMPLE: Brewed Coffee, regular	● 12 16 20 24	0 1 ● 3 4 5 6 7 8	● W M Y	1 1
Brewed Coffee (hot or cold)............................	8 12 16 20 24	0 1 2 3 4 5 6 7 8	D W M Y	2 2
Espresso..	8 12 16 20 24	0 1 2 3 4 5 6 7 8	D W M Y	3 3
Cappuccino...	8 12 16 20 24	0 1 2 3 4 5 6 7 8	D W M Y	4 4
Frozen blended coffee drink...........................	8 12 16 20 24	0 1 2 3 4 5 6 7 8	D W M Y	5 5
Hot brewed Tea..	8 12 16 20 24	0 1 2 3 4 5 6 7 8	D W M Y	6 6
Iced Tea..	8 12 16 20 24	0 1 2 3 4 5 6 7 8	D W M Y	7 7
Other ______________________________	8 12 16 20 24	0 1 2 3 4 5 6 7 8	D W M Y	8 8
**SOFT DRINK (Regular and diet)**				9 9
Cola-Type	8 12 16 20 24	0 1 2 3 4 5 6 7 8	D W M Y	0 0
Coke Blak...	8 12 16 20 24	0 1 2 3 4 5 6 7 8	D W M Y	1 1
Pepper-Type (Dr. Pepper, Mr. PIBB, etc)......	8 12 16 20 24	0 1 2 3 4 5 6 7 8	D W M Y	2 2
Tab Energy...	8 12 16 20 24	0 1 2 3 4 5 6 7 8	D W M Y	3 3
Mountain Dew/Mello Yello...........................	8 12 16 20 24	0 1 2 3 4 5 6 7 8	D W M Y	4 4
Jolt or Vault Energy drinks............................	8 12 16 20 24	0 1 2 3 4 5 6 7 8	D W M Y	5 5
Sunkist Orange Soda (only this brand)...........	8 12 16 20 24	0 1 2 3 4 5 6 7 8	D W M Y	6 6
Barq's Root Beer (regular only/just this brand)	8 12 16 20 24	0 1 2 3 4 5 6 7 8	D W M Y	7 7
Other ______________________________	8 12 16 20 24	0 1 2 3 4 5 6 7 8	D W M Y	8 8
**OTHER BEVERAGES (Regular and diet)**	Number of cans/bottles			9 9
Glaceau Vitaminwater Energy Tropical..........	1 2 3 4 5 6	0 1 2 3 4 5 6 7 8	D W M Y	0 0
AMP Energy Drink...	1 2 3 4 5 6	0 1 2 3 4 5 6 7 8	D W M Y	1 1
Red Bull Energy Drink..................................	1 2 3 4 5 6	0 1 2 3 4 5 6 7 8	D W M Y	2 2
Elements Atomic Jacked Apple Juice.............	1 2 3 4 5 6	0 1 2 3 4 5 6 7 8	D W M Y	3 3
SoBe Energy Citrus	1 2 3 4 5 6	0 1 2 3 4 5 6 7 8	D W M Y	4 4
SoBe adrenaline sport drink...........................	1 2 3 4 5 6	0 1 2 3 4 5 6 7 8	D W M Y	5 5
Fruit2O Plus Energy......................................	1 2 3 4 5 6	0 1 2 3 4 5 6 7 8	D W M Y	6 6
PJ Tight/Pimp Juice.....................................	1 2 3 4 5 6	0 1 2 3 4 5 6 7 8	D W M Y	7 7
BAWLS Guarana...	1 2 3 4 5 6	0 1 2 3 4 5 6 7 8	D W M Y	8 8
Monster Energy Drink..................................	1 2 3 4 5 6	0 1 2 3 4 5 6 7 8	D W M Y	9 9
Rock Star Energy Drink................................	1 2 3 4 5 6	0 1 2 3 4 5 6 7 8	D W M Y	0 0
Other ______________________________	1 2 3 4 5 6	0 1 2 3 4 5 6 7 8	D W M Y	1 1
**GUM/MEDICATIONS**	Number of pills/sticks of gum			2 2
Jolt gum...	1 2 3 4 5 6	0 1 2 3 4 5 6 7 8	D W M Y	3 3
Stay Alert gum...	1 2 3 4 5 6	0 1 2 3 4 5 6 7 8	D W M Y	4 4
Vivarin/NoDoz Maximum............................	1 2 3 4 5 6	0 1 2 3 4 5 6 7 8	D W M Y	5 5
NoDoz regular/Generic Caffeine pills...........	1 2 3 4 5 6	0 1 2 3 4 5 6 7 8	D W M Y	6 6
Dexatrim or other weight control aids...........	1 2 3 4 5 6	0 1 2 3 4 5 6 7 8	D W M Y	7 7
Bayer Headache Relief/Excedrin pills.........	1 2 3 4 5 6	0 1 2 3 4 5 6 7 8	D W M Y	8 8
Other ________________________________	1 2 3 4 5 6	0 1 2 3 4 5 6 7 8	D W M Y	9 9

34. Based on the past six months, use the table to estimate your use of each of the following vitamins, minerals, and/or supplements. For the next three pages, please fill in one circle for each item, then record the reason for use. DO NOT list the vitamins found in your MultiVitamin or Antioxidant under the individual vitamin and mineral section, unless there is a "Plus" ingredient added, such as Multi Vitamin and Minerals plus Iron. Then, you would fill out Multi Vitamin AND Iron.

	Estimation of use					Reason for use							
	NEVER	ONCE A MONTH	ONCE A WEEK	FEW TIMES/WEEK (2-6 TIMES)	DAILY	PERFORMANCE ENHANCER	PROMOTE GENERAL HEALTH	GIVE MORE ENERGY	WEIGHT LOSS	INCREASED ENDURANCE	GREATER MUSCLE STRENGTH	NOT SURE	OTHER*
Multiple Vitamin Supplement	0	1	2	3	4	1	2	3	4	5	6	7	8
Mega/High Potency Vitamin	0	1	2	3	4	1	2	3	4	5	6	7	8
Combination Antioxidant Supplement	0	1	2	3	4	1	2	3	4	5	6	7	8
**Individual Vitamins/Minerals**													
Vitamin A	0	1	2	3	4	1	2	3	4	5	6	7	8
Vitamin C	0	1	2	3	4	1	2	3	4	5	6	7	8
Vitamin D	0	1	2	3	4	1	2	3	4	5	6	7	8
Vitamin E	0	1	2	3	4	1	2	3	4	5	6	7	8
Beta-carotene	0	1	2	3	4	1	2	3	4	5	6	7	8
B-Complex Supplement	0	1	2	3	4	1	2	3	4	5	6	7	8
B2 (Riboflavin) alone	0	1	2	3	4	1	2	3	4	5	6	7	8
B5 (Pantothenic acid) alone	0	1	2	3	4	1	2	3	4	5	6	7	8
B6 (Pyridoxine) alone	0	1	2	3	4	1	2	3	4	5	6	7	8
B12 (Cyanocobalamin) alone	0	1	2	3	4	1	2	3	4	5	6	7	8
Calcium	0	1	2	3	4	1	2	3	4	5	6	7	8
Chromium	0	1	2	3	4	1	2	3	4	5	6	7	8
Folate (Folic acid, Folacin)	0	1	2	3	4	1	2	3	4	5	6	7	8
Iron	0	1	2	3	4	1	2	3	4	5	6	7	8
Magnesium	0	1	2	3	4	1	2	3	4	5	6	7	8
Phosphate (Phosphorous)	0	1	2	3	4	1	2	3	4	5	6	7	8
Potassium	0	1	2	3	4	1	2	3	4	5	6	7	8
Selenium	0	1	2	3	4	1	2	3	4	5	6	7	8
Zinc	0	1	2	3	4	1	2	3	4	5	6	7	8
Other:_____________	0	1	2	3	4	1	2	3	4	5	6	7	8
Other:_____________	0	1	2	3	4	1	2	3	4	5	6	7	8
Other:_____________	0	1	2	3	4	1	2	3	4	5	6	7	8
Other:_____________	0	1	2	3	4	1	2	3	4	5	6	7	8

***Please explain your response to OTHER REASON FOR USE**

____

____

1 2 3 4 5 6 7 8 9 10 11 12 13 14 15 16 17 18

0 1 2 3 4 5 6 7 8 9	Q34_OTH1
0 1 2 3 4 5 6 7 8 9	Q34_OTH2
0 1 2 3 4 5 6 7 8 9	Q34_OTH3
0 1 2 3 4 5 6 7 8 9	Q34_OTH4

Dietary Supplement Survey

35. Protein and Other Supplements

	Estimation of use: NEVER	ONCE A MONTH	ONCE A WEEK	FEW TIMES/WEEK (2-6 TIMES)	DAILY	Reason for use: PERFORMANCE ENHANCER	PROMOTE GENERAL HEALTH	GIVE MORE ENERGY	WEIGHT LOSS	INCREASED ENDURANCE	GREATER MUSCLE STRENGTH	NOT SURE	OTHER*
Protein Powder	0	1	2	3	4	1	2	3	4	5	6	7	8
Amino Acid Mixtures	0	1	2	3	4	1	2	3	4	5	6	7	8
Arginine/Nitric Oxide - alone	0	1	2	3	4	1	2	3	4	5	6	7	8
Creatine - alone	0	1	2	3	4	1	2	3	4	5	6	7	8
Glutamine - alone	0	1	2	3	4	1	2	3	4	5	6	7	8
Other_______________	0	1	2	3	4	1	2	3	4	5	6	7	8
**OTHER SUPPLEMENTS**													
Alpha lipoic acid	0	1	2	3	4	1	2	3	4	5	6	7	8
Androstenedione (andro)	0	1	2	3	4	1	2	3	4	5	6	7	8
BCAA (branch chain AA)	0	1	2	3	4	1	2	3	4	5	6	7	8
Caffeine	0	1	2	3	4	1	2	3	4	5	6	7	8
CoQ 10 (CoEnzyme Q10)	0	1	2	3	4	1	2	3	4	5	6	7	8
DHEA	0	1	2	3	4	1	2	3	4	5	6	7	8
Ephedrine/ephedra	0	1	2	3	4	1	2	3	4	5	6	7	8
Garlic	0	1	2	3	4	1	2	3	4	5	6	7	8
Ginkgo Biloba	0	1	2	3	4	1	2	3	4	5	6	7	8
Ginseng	0	1	2	3	4	1	2	3	4	5	6	7	8
Glucosomine/Chondroitin	0	1	2	3	4	1	2	3	4	5	6	7	8
Guarana	0	1	2	3	4	1	2	3	4	5	6	7	8
Herbal Phentermine	0	1	2	3	4	1	2	3	4	5	6	7	8
Hoodia Gordonii	0	1	2	3	4	1	2	3	4	5	6	7	8
Horney goat weed	0	1	2	3	4	1	2	3	4	5	6	7	8
L-Carnitine	0	1	2	3	4	1	2	3	4	5	6	7	8
Lycopene	0	1	2	3	4	1	2	3	4	5	6	7	8
Meal replacement drinks	0	1	2	3	4	1	2	3	4	5	6	7	8
Melatonin	0	1	2	3	4	1	2	3	4	5	6	7	8
Psyllium	0	1	2	3	4	1	2	3	4	5	6	7	8
Sports Bars (Powerbar, Tiger's Milk)	0	1	2	3	4	1	2	3	4	5	6	7	8
Sports jelly beans	0	1	2	3	4	1	2	3	4	5	6	7	8
Sports drinks (Gatorade, Powerade)	0	1	2	3	4	1	2	3	4	5	6	7	8
Sports gels	0	1	2	3	4	1	2	3	4	5	6	7	8
Synephrine/Bitter Orange	0	1	2	3	4	1	2	3	4	5	6	7	8
Tyrosine	0	1	2	3	4	1	2	3	4	5	6	7	8
Willow bark	0	1	2	3	4	1	2	3	4	5	6	7	8
Yerba mate	0	1	2	3	4	1	2	3	4	5	6	7	8
Yohimbe	0	1	2	3	4	1	2	3	4	5	6	7	8

***Please explain your response to OTHER REASON FOR USE**

____

0 1 2 3 4 5 6 7 8 9	Q35_OTH1
0 1 2 3 4 5 6 7 8 9	Q35_OTH2
0 1 2 3 4 5 6 7 8 9	Q35_OTH3
0 1 2 3 4 5 6 7 8 9	Q35_OTH4

1 2 3 4 5 6 7 8 9 10 11 12 13 14 15 16 17 18

36. Specific brand of supplements. If your brand is not listed, please write it in the OTHER line and fill out the appropriate use information.

	Estimation of use					Reason for use							
	NEVER	ONCE A MONTH	ONCE A WEEK	FEW TIMES/WEEK (2-6 TIMES)	DAILY	PERFORMANCE ENHANCER	PROMOTE GENERAL HEALTH	GIVE MORE ENERGY	WEIGHT LOSS	INCREASED ENDURANCE	GREATER MUSCLE STRENGTH	NOT SURE	OTHER*
Enzyte	0	1	2	3	4	1	2	3	4	5	6	7	8
Exitor	0	1	2	3	4	1	2	3	4	5	6	7	8
Fizogen On Cycle	0	1	2	3	4	1	2	3	4	5	6	7	8
GNC Fish Body Oils	0	1	2	3	4	1	2	3	4	5	6	7	8
GNC's DHEA Vitapak	0	1	2	3	4	1	2	3	4	5	6	7	8
GNC's Performance and Vitality Program	0	1	2	3	4	1	2	3	4	5	6	7	8
GNC's Triflex	0	1	2	3	4	1	2	3	4	5	6	7	8
Herbal Clean Qcarbo	0	1	2	3	4	1	2	3	4	5	6	7	8
Inositol	0	1	2	3	4	1	2	3	4	5	6	7	8
Instone Lean Fire	0	1	2	3	4	1	2	3	4	5	6	7	8
Lipitrex	0	1	2	3	4	1	2	3	4	5	6	7	8
Magna RX	0	1	2	3	4	1	2	3	4	5	6	7	8
Maxx Trim	0	1	2	3	4	1	2	3	4	5	6	7	8
MHP T-Bomb II	0	1	2	3	4	1	2	3	4	5	6	7	8
MRI NO2 Hemodilator	0	1	2	3	4	1	2	3	4	5	6	7	8
MuscleTech Cell-Tech	0	1	2	3	4	1	2	3	4	5	6	7	8
MuscleTech Hydroxycut	0	1	2	3	4	1	2	3	4	5	6	7	8
MuscleTech Nitro-Tech	0	1	2	3	4	1	2	3	4	5	6	7	8
MuscleTech Pump-Tech	0	1	2	3	4	1	2	3	4	5	6	7	8
NxCare Anavol	0	1	2	3	4	1	2	3	4	5	6	7	8
NxCare Nitro T3	0	1	2	3	4	1	2	3	4	5	6	7	8
Pinnacle NOX2	0	1	2	3	4	1	2	3	4	5	6	7	8
Preventive Nutrition Heart Advance	0	1	2	3	4	1	2	3	4	5	6	7	8
Preventive Nutrition Triple Cleanse	0	1	2	3	4	1	2	3	4	5	6	7	8
Prolab Cuts II	0	1	2	3	4	1	2	3	4	5	6	7	8
Prolab Metabolic Thyrolean	0	1	2	3	4	1	2	3	4	5	6	7	8
Prolab N-Large2	0	1	2	3	4	1	2	3	4	5	6	7	8
Pro-Performance CLA	0	1	2	3	4	1	2	3	4	5	6	7	8
Pro-Performance Thermoburst	0	1	2	3	4	1	2	3	4	5	6	7	8
Pro-Performance Thermogain	0	1	2	3	4	1	2	3	4	5	6	7	8
Royal Perfection Recovery	0	1	2	3	4	1	2	3	4	5	6	7	8
TrimSpa	0	1	2	3	4	1	2	3	4	5	6	7	8
Twin Labs Amino Fuel	0	1	2	3	4	1	2	3	4	5	6	7	8
Twin Labs Ripped Fuel	0	1	2	3	4	1	2	3	4	5	6	7	8
Xenadrine	0	1	2	3	4	1	2	3	4	5	6	7	8
Zantrex	0	1	2	3	4	1	2	3	4	5	6	7	8

***Please explain your response to OTHER REASON FOR USE**

____

0 1 2 3 4 5 6 7 8 9	Q36_OTH1
0 1 2 3 4 5 6 7 8 9	Q36_OTH2
0 1 2 3 4 5 6 7 8 9	Q36_OTH3
0 1 2 3 4 5 6 7 8 9	Q36_OTH4

1 2 3 4 5 6 7 8 9 10 11 12 13 14 15 16 17 18

Dietary Supplement Survey

37. Other Supplements
Please list any other supplements that you may be currently taking.

	Estimation of use						Reason for use							
	NEVER	ONCE A MONTH	ONCE A WEEK	FEW TIMES/WEEK (2-6 TIMES)	DAILY		PERFORMANCE ENHANCER	PROMOTE GENERAL HEALTH	GIVE MORE ENERGY	WEIGHT LOSS	INCREASED ENDURANCE	GREATER MUSCLE STRENGTH	NOT SURE	OTHER*
Other________________________	0	1	2	3	4		1	2	3	4	5	6	7	8
Other________________________	0	1	2	3	4		1	2	3	4	5	6	7	8
Other________________________	0	1	2	3	4		1	2	3	4	5	6	7	8
Other________________________	0	1	2	3	4		1	2	3	4	5	6	7	8
Other________________________	0	1	2	3	4		1	2	3	4	5	6	7	8
Other________________________	0	1	2	3	4		1	2	3	4	5	6	7	8
Other________________________	0	1	2	3	4		1	2	3	4	5	6	7	8

***Please explain your response to OTHER REASON FOR USE**

____

____

1 2 3 4 5 6 7 8 9 10 11 12 13 14 15 16 17 18

0 1 2 3 4 5 6 7 8 9	Q37_OTH1
0 1 2 3 4 5 6 7 8 9	Q37_OTH2
0 1 2 3 4 5 6 7 8 9	Q37_OTH3
0 1 2 3 4 5 6 7 8 9	Q37_OTH4
0 1 2 3 4 5 6 7 8 9	Q37_OTH5
0 1 2 3 4 5 6 7 8 9	Q37_OTH6
0 1 2 3 4 5 6 7 8 9	Q37_OTH7

38. How confident are you that your dietary supplements will do as they claim?

1 Extremely confident
2 Very confident
3 Somewhat confident
4 Not confident at all

39. How confident are you that your dietary supplements are safe to consume?

1 Extremely confident
2 Very confident
3 Somewhat confident
4 Not confident at all

40. Have you experienced any of the following negative side effects while consuming dietary supplements?
(Mark all that apply)

1 Abnormal rapid heart beat
2 Stomach pain
3 Dizziness or confusion
4 Tremors or shaking
5 Numbness or tingling of arms or legs
6 Loss of consciousness
7 Other_______________
8 I did not experience any negative side effects

Do not write in this box
0 1 2 3 4 5 6 7 8 9
0 1 2 3 4 5 6 7 8 9
Q40_oth

41. What or who is the source of your dietary supplement information? (Mark all that apply)

1 My recruiter
2 Family members
3 Friends
4 A Health professional (such as a doctor, nurse, dietitian, etc)
5 A personal trainer
6 Magazines (such as Mens Health, Muscle and Fitness, Flex, Shape, and SELF)
7 Books
8 Peer review journal (such as Medicine and Science in Sports and Exercise; International Journal of Sports Medicine)
9 Internet
10 Store sales person
11 Television
12 Other:_______________________________

Do not write in this box
0 1 2 3 4 5 6 7 8 9
0 1 2 3 4 5 6 7 8 9
Q41_oth

42. Where do you purchase your supplements? (Mark all that apply)

1 I do not use supplements
2 On post GNC store
3 Off post GNC store
4 GNC website
5 Other supplement store (such as Vitamin World, Vitamin Cottage)
6 AAFES PX/BX (do not include GNC)
7 Drug store (such as CVS, Walgreens, Eckerd)
8 Grocery store
9 Health food store (such as Wild Oats or Whole foods)
10 Gym/Fitness center
11 Not Sure
12 Other Store: __________________________
13 Other Internet Site:_______________________________
14 Other Source:_________________________

Do not write in this box
0 1 2 3 4 5 6 7 8 9
0 1 2 3 4 5 6 7 8 9
Q42_store
0 1 2 3 4 5 6 7 8 9
0 1 2 3 4 5 6 7 8 9
Q42Internet
0 1 2 3 4 5 6 7 8 9
0 1 2 3 4 5 6 7 8 9
Q42_oth

43. During the past three months, on average, how much money did you spend per month on dietary supplements:

Whole dollar amount
0 1 2 3 4 5 6 7 8 9
0 1 2 3 4 5 6 7 8 9
0 1 2 3 4 5 6 7 8 9

Thank you for taking the time to fill out this survey.
Please check over the questions to be sure nothing was missed.

PLEASE DO NOT WRITE IN THIS AREA

[SERIAL]
